# Chopping Roughage Length Improved Rumen Development of Weaned Calves as Revealed by Rumen Fermentation and Bacterial Community

**DOI:** 10.3390/ani10112149

**Published:** 2020-11-19

**Authors:** Haibo Wang, Fei Wu, Tianci Guan, Yangxiang Zhu, Zhantao Yu, Depeng Zhang, Siyu Zhang, Huawei Su, Binghai Cao

**Affiliations:** 1 State Key Laboratory of Animal Nutrition, College of Animal Science and Technology, China Agricultural University, Beijing 100193, China; wanghb630@163.com (H.W.); F_Wu1995@163.com (F.W.); Guan_tianci@163.com (T.G.); chinazhuyx@163.com (Y.Z.); zhangdepeng@cau.edu.cn (D.Z.); zsycaucast@163.com (S.Z.); 2 Key Laboratory of Qinghai-Tibetan Plateau Animal Genetic Resource Reservation and Utilization, Southwest Minzu University, Ministry of Education, Chengdu 610000, China; 3 Department of Animal Science, University of Tennessee, Knoxville, TN 37996, USA; zyu18@vols.utk.edu

**Keywords:** roughage particle size, nutrient digestibility, rumen fermentation, plasma parameters, rumen bacterial community

## Abstract

**Simple Summary:**

The development of rumen plays vital roles on the growth performance of calves. Rumen development is determined by rumen microorganisms, their metabolic products, and diet. Volatile fatty acids produced by ruminal microbes are one of the major determinants of ruminal papillary size and shape. Feed particle size could affect the development of rumen. Whether the roughage length determines the rumen development through the rumen microflora or not is worthy of investigation. The aim of this study was to investigate the comprehensive effects of roughage length and rumen bacterial community on the rumen development of weaned calves. Our results indicated that chopping roughage increased the ruminal bacteria involved in increasing the production of butyrate and promoted the process of bacterial colonization. This study illustrated chopping roughage at a short length could improve the rumen development and promote a stable rumen bacterial community of weaned calves.

**Abstract:**

Roughage particle size can influence rumen development, which is also determined by rumen microorganisms and their metabolic end-products. Therefore, the aim of this study was to evaluate the comprehensive effects of roughage length and rumen bacterial community on the rumen development of weaned calves. A total of thirty-six weaned Angus female calves (125 ± 3 d; 161.2 ± 13.0 kg) were randomly assigned to three diets differing in roughage particle size: 4 cm (short length); 24 cm (medium length); and 44 cm (long length). Results showed that chopping roughage increased dry matter intake and organic matter apparent digestibility; altered rumen fermentation indicated by the increased rumen butyrate and valerate concentrations; and increased plasma glucose, cholesterol, and total protein. Chopping roughage affected rumen bacterial community, as indicated by altering the diversity indices; by increasing ruminal bacteria *Papillibacter* and *Eubacterium_hallii_group*, which are involved in butyrate production; and by increasing *Synergistetes* and *Mogibacterium*, which are involved in bacterial colonization. In conclusion, chopping roughage at 4 cm was shown to improve the rumen bacterial community, alter rumen fermentation, eventually promote the development of rumen.

## 1. Introduction

Beef calves are generally weaned by the abrupt separation of cows and calves, as well as changes in feed types and environment, which eventually cause weaning stress in calves [1]. Weaning stress plays vital roles in affecting the subsequent health and growth performance of calves. The physical characteristics of a diet could affect forestomach anatomical and microbial development, which can further influence the future performance of calves [2]. Typically, young calves are usually fed with starter diets with high concentrate proportions in order to promote the development of rumen [3]. The fiber source is necessary for young calves due to its benefic functions, including stimulating rumination, expanding rumen volume, and reducing the keratinization of rumen papillae [4].

Roughage could affect the rate of ruminal acid production and dry matter intake (DMI) through various mechanisms, including altering chewing, rumination, and ruminal digesta kinetics [5]. Pelleted starter diets with a low neutral detergent fiber (NDF) are often recommended for the preweaning calves, while the provision of roughage is urgent after weaning [6]. Chopping roughage length is generally considered to increase DMI owing to the shorter rumination time, faster passage rate in the rumen [7], and less wastage by avoiding selective consumption [8]. However, a fast passage rate in the rumen and shorter rumination time may result in reducing the ruminal pH due to limited buffer capacity [9]. Many researchers have studied the effect of roughage particle size on the rumen fermentation and growth of calves. Nemati et al. found that increasing the particle size of alfalfa hay from 1 to 3 mm improved calf performance when calves were fed finely ground starter feeds [10]. Mirzaei et al. showed that calves fed roughage with the particle size of 5.04 mm increased starter intake and weaning weight compared the particle size of 2.92 mm [11]. Recently, researchers found that increasing the particle size of straw from 3.04 to 12.7 mm caused little impact on ruminal fermentation [12]. Hence, the longer particle size of roughage needs to be further explored to investigate the rumen development of weaned calves.

The rumen plays critical roles in the ruminant production systems due to the large amount of microorganism colonization. Bacteria, as the predominant abundant and diverse community of ruminal microbes, play roles in fermenting plant polysaccharides and protein to generate nutrients for the maintenance and growth of the host [13]. The fermentation products of microorganism are mainly the volatile fatty acids (VFA), which have a variety functions, including providing an energy source for the host and stimulating the development of rumen [14]. A shorter particle size has been described to decrease rumen pH, inhibit rumen microbial activity, and reduce the feed digestion rate and apparent digestibility of lactating cows [15]. Limited prior literature has been involved in evaluating the effects of roughage length on the rumen microflora of weaned calves. Therefore, the purpose of this study was to investigate the effects of the roughage length on the rumen fermentation and bacterial microbiome of weaned calves.

## 2. Material and Methods

### 2.1. Treatments, Animals, and Experimental Design

The study was carried out in Inner Mongolia Lvfengquan Agriculture and Animal Husbandry Technology Co., Ltd. (Hinggan, Inner Mongolia, China), whose experimental procedures had been approved by the Animal Welfare and Ethics Committee of China Agricultural University (AW13012020-1). Thirty-six weaned Angus female calves (age: 125 ± 3 d; live weight: 161.2 ± 13.0 kg) were randomly allocated into three dietary groups according to body weight, with each group composed of 12 replications and one calf for each replication. The oat hay, as the sole roughage, was measured using a ruler and chopped using a hand hay cutter to achieve the length of 4 cm (short length, SL), 24 cm (medium length, ML), and 44 cm (long length, LL). The starter pellets were purchased from Purina (Cargill Feed Co., Ltd., Jilin, China). The dietary ingredients and chemical compositions are shown in Table 1. Calves were offered the starter pellets equally and allowed ad libitum to feed on roughage. The experimental period was 40 days; feed and orts were recorded daily, and the dry matter contents of the pellets and roughage were measured weekly, then DMI was calculated. The calves’ weights were recorded at day 0 and day 34 to calculate the average daily gain (ADG). The digestion experiment was conducted from day 35 to day 38; feed and feces samples were collected on three consecutive days, a quarter of feces weight of 10% tartaric acid solution was mixed with feces, then dried at 65 °C for 48 h for further analysis. Blood samples were collected from tail vein into a vacuum tube containing heparin sodium before morning feeding at day 40, then plasma was separated after the blood was centrifuged at 3000× *g* for 10 min.

At the end of the experiment, the rumen fluid was collected at 3 h after morning feeding using an esophageal tube, which was cleaned thoroughly with fresh water during the samples collection. After discarding the first 50 mL of rumen fluid to minimize saliva contamination, then collecting about 50 mL of rumen fluid (both solid and liquid fractions), the pH of the rumen fluid was immediately measured using a portable pH meter (HJ-90B, Aerospace Electronics, Beijing, China). Rumen fluid samples were handled in two ways. The first method involved transferring about 2 mL of rumen fluid into an internal thread cryogenic vial and immediately freezing it with liquid nitrogen. Four rumen fluid samples were randomly selected from each group; thus, a total of 12 rumen fluid samples were used to perform the high-sequencing analysis. The second method involved centrifuging the rumen fluid at 3000× *g* for 10 min and taking the supernatant; then, 1 mL of supernatant was mixed with 0.25 mL of metaphosphoric acid standard solution (25 g/100 mL) for the determination of VFA and NH_3_-N.

### 2.2. Chemical Analysis

The dry matter (DM), ash, crude protein (CP), and ether extract (EE) of feed and fecal samples were measured with the AOAC method [16]. Organic matter (OM) content was calculated as 100% minus the ash content. The CP and EE were analyzed using a distiller Kjeldahl (Kjeltec 2100, Foss, Hillerød, Denmark) and an AnkomXT15 Extractor (Ankom Technology, Fairport, NY, USA), respectively. The NDF and acid detergent fiber (ADF) of feed and fecal samples were measured using an Ankom fiber analyzer (Ankom Technology) following Van Soest’s method [17]. The acid-insoluble ash (AIA) was used as the endogenous tracer to analysis the nutrient apparent digestibility according the procedure reported by Van Keulen and Young [18], with the formula depicted by Zhong et al. [19], with the formula as follows:

D = [1 − (Ad × Nf)/(Af × Nd)] × 100

where D represents the digestibility of the nutrient; Ad (g/kg) and Af (g/kg) represent the AIA of the diet and feces, respectively; and Nd (g/kg) and Nf (g/kg) represent the nutrient contents of the diet and feces, respectively.

### 2.3. Rumen Fermentation Parameters

The NH_3_-N of rumen fluid was measured by alkaline phenol hypochlorite method [20]. The VFA of rumen fluid was measured using a gas chromatograph (GC-2014 Shimadzu Corporation, Kyoto, Japan) according to the method described by Wang et al. [21]. In short, the column nitrogen flow rate was controlled at 46.3 cm/s, and the injection volume of rumen fluid was 0.4 μL. The inlet temperature and detector temperature were maintained at 220 °C and 250 °C, respectively. The column temperature was initially set at 110 °C and held for 30 s, then increased to 120 °C at 10 °C/min and held for 4 min, and then subsequently heated up to 150 °C at 10 °C/min.

### 2.4. Plasma Biochemical Parameters

Plasma biochemical indicators were analyzed using a clinical autoanalyzer (Hitachi 7020; Hitachi Co., Tokyo, Japan) using commercial test kits (Beijing Strong Biotechnologies, Inc., Beijing, China). Plasma insulin (INS), growth hormone (GH), and insulin-like growth factor 1 (IGF1) were measured according to the instructions of ELISA kits (Shanghai Enzyme-linked Biotechnology Co., Ltd., Shanghai, China).

### 2.5. DNA Extraction and 16S rRNA Pyrosequencing

Ruminal fluid DNA samples were extracted using a kit (Omega Bio-Tek, Norcross, GA, USA). DNA concentration and purity were measured using the Nanodrop 2000 spectrophotometer (Technologies Inc., Wilmington, DE, USA). The V3 and V4 hypervariable regions were amplified with the following primers: 338F and 806R. A 25 μL reaction system was performed as follows: 2× Taq PCR MasterMix (12.5 μL), BSA (3 μL; 2 ng/μL), Primer (2 μL; 5 μM), template DNA (2 μL), and RNase-free ddH_2_O (5.5 μL). Cycling parameters were as follows: 95 °C for 5 min; followed by 32 cycles of 95 °C for 45 s, 55 °C for 50 s, and 72 °C for 45 s; and, finally, 72 °C for 10 min. The three PCR products of each sample were combined to reduce the PCR deviation of the reaction level and purify, quantify, and sequence the PCR products. Then, the Illumina MiSeq platform (San Diego, CA, USA) was used to obtain the paired-end sequences.

The raw sequences were filtered according the following situations: the sequences length was less than 200 bp; sequences contained ambiguous bases and chimera; sequences had a quality score below 20. Based on 97% similarity, the sequences were clustered into operational taxonomies units (OTUs). Sample-specific barcode sequences were used to separate qualified reads and trimmed using Illumina Analysis Pipeline version 2.6 (Illumina, San Diego, CA, USA). Then, Quantitative Analysis of Microbial Ecology (QIIME, http://qiime.org/) was used to analyze the dataset. Alpha diversity indexes were calculated using Mothur (Patrick Schloss, Ann Arbor, MI, USA). Taxonomy classifications for each OTU were obtained by assigning against the Silva bacterial alignment database according to the Ribosomal Database Project classifier tool (http://sourceforge.net/projects/rdp-classifier/).

The raw sequencing data were deposited to Sequence Read Archive (SRA) under the accession number PRJNA602042.

### 2.6. Statistical Analysis

The MIXED procedures of SAS 9.0 (SAS Inst. Inc., Cary, NC, USA) were taken to analyze the data, and the effects of roughage length (SL, ML, and LL) were evaluated using the linear or quadratic comparisons. The Pearson rank correlation test in SAS was used to analyze the rumen fermentation parameters, nutrient digestibility, and rumen bacterial abundance. Significant differences were declared at *p* < 0.05, and extremely significant differences were declared at *p* < 0.01.

## 3. Results

### 3.1. Growth Performance and Nutrient Apparent Digestibility

The growth performance and nutrient apparent digestibility of weaned Angus calves are shown in Table 2. Chopping roughage had no significant effects on the final body weight, ADG, and feed conversion ratio (FCR) of weaned calves (*p* > 0.05). The oat hay intake and the roughage ratio were increased linearly and quadratically by chopping roughage. The OM apparent digestibility was quadratically affected by chopping roughage. The apparent digestibility of the CP, EE, NDF, and ADF was not affected by chopping roughage (*p* > 0.05).

### 3.2. Rumen Fermentation Characteristics

As shown in Table 3, chopping roughage had no effects on the pH and NH_3_-N concentrations in ruminal fluid (*p* > 0.05). Chopping roughage linearly and quadratically increased the concentrations of butyrate and valerate; besides, there was a linear trend for isobutyrate (*p* = 0.062). There were no differences for the concentrations of ruminal fluid total VFA, acetate, propionate, and the ratio of acetate to propionate (*p* > 0.05).

### 3.3. Plasma Parameters

The effects of roughage length on plasma parameters are shown in Table 4. Chopping roughage linearly increased the concentration of plasma glucose, and linearly and quadratically increased the concentrations of cholesterol and total protein. No significant difference in plasma triglyceride, urea nitrogen, INS, GH, and IGF1 concentrations was found among the treatments (*p* > 0.05).

### 3.4. Sequencing Depth and Diversity Estimates

A total of 229,680 clean tags were retained after quality control from the 12 samples, with an average of 19,140 per sample. These high-quality sequences were distributed with an average length of 420 bp, based on a 97% similarity cut-off, were assigned to 1379 OTUs of rumen bacteria. Chopping roughage linearly and quadratically decreased chao 1 and richness estimator PD_whole_tree, and quadratically decreased the observed_species (Table 5).

### 3.5. Rumen Bacteria Composition

**Figure 1:**
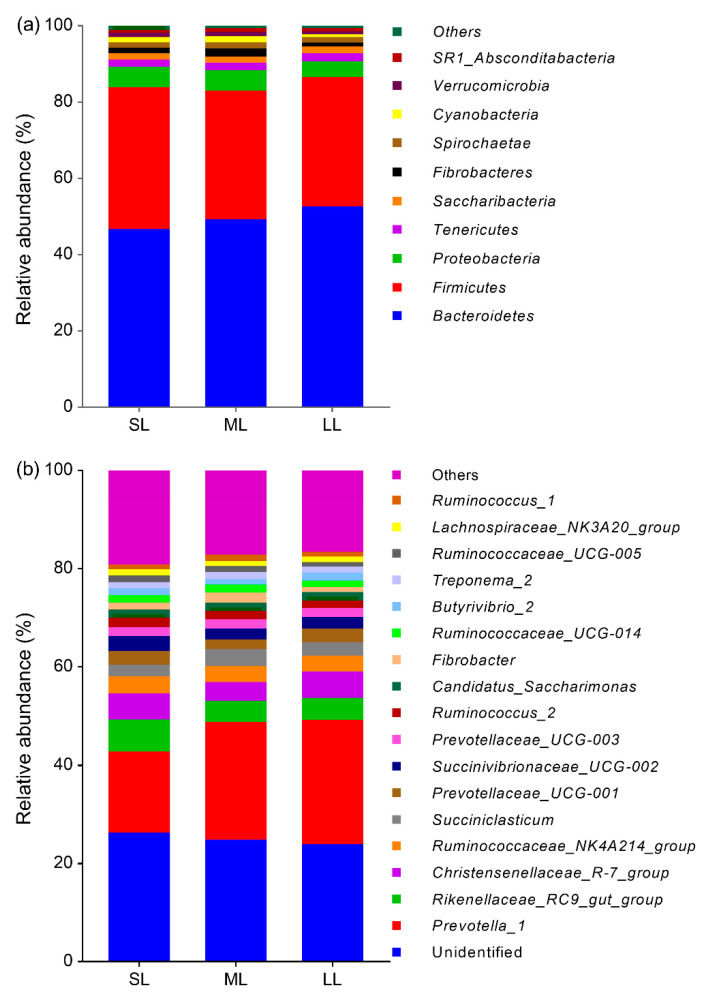
Effects of roughage length on ruminal bacteria at phyla (**a**) and genus (**b**) levels. SL: short length; ML: medium length; LL: long length.

There were 22 identified phyla of rumen bacteria and only phylum percentages exceeding 1% are listed in Figure 1a. Among them, Bacteroidetes (48.97%) and Firmicutes (34.91%) were the dominant phyla, followed by Proteobacteria (5.00%), Tenericutes (1.95%), Saccharibacteria (1.67%), Fibrobacteres (1.54%), Spirochaete (1.49%), and Cyanobacteria (1.23%); minor phyla included *Verrucomicrobia* (0.99%) and SR1_Absconditabacteria (0.90%). There were 215 identified genera of rumen bacteria and only genus percentages exceeding 1% are listed in Figure 1b. Among them, *Prevotella_1* (21.88%) was the most abundant genus, followed by *Rikenellaceae_RC9_gut_group* (5.05%), *Christensenellaceae_R-7_group* (4.85%), *Ruminococcaceae_NK4A214_group* (3.34%), *Succiniclasticum* (2.82%), *Prevotellaceae_UCG-001* (2.54%) and *Succinivibrionaceae_UCG-002* (2.51%), and 24.60% of the sequences were unidentified.

As shown in Table S1, chopping roughage linearly and quadratically increased the relative abundance of the phylum Synergistetes. At the genus level, chopping roughage linearly increased the relative abundances of *Ruminococcaceae_*UCG-005 and *Papillibacter*, quadratically increased the relative abundances of *Family_XIII_AD3011_group*, *Eubacterium_hallii_group* and *Mogibacterium*, and linearly and quadratically increased the relative abundances of *Prevotellaceae_*UCG-004 and *Pyramidobacter*. The relative abundances of *Succinivibrio* and *Selenomonas_1* were quadratically decreased by chopping roughage (Table S2).

### 3.6. Correlation Analysis

**Figure 2:**
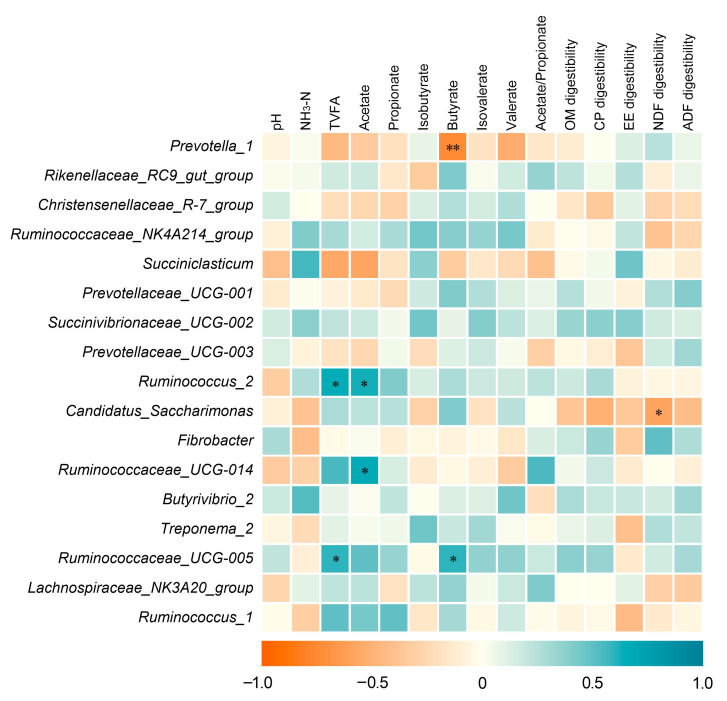
Correlation between fermentation products, nutrient digestibility, and rumen bacteria. The genera representing at least 1% of the bacterial community are presented. The blue lattices denote that the correlation is positive (closer to 1), and the red lattices denote that the correlation is negative (closer to −1) between ruminal bacteria and fermentation parameters. * *p* < 0.05; ** *p* < 0.01.

The correlation between rumen fermentation products, nutrient digestibility, and rumen bacteria is shown in Figure 2. The relative abundances of *Ruminococcus_*2 (*r* = 0.656, *p* = 0.021) and *Ruminococcaceae_*UCG-005 (*r* = 0.594, *p* = 0.042) positively correlated with the concentration of total VFA. The relative abundances of *Ruminococcus_*2 (*r* = 0.621, *p* = 0.031) and *Ruminococcaceae_UCG-014* (*r* = 0.662, *p* = 0.019) positively correlated with the concentration of acetate. The relative abundance of *Ruminococcaceae_UCG-005* positively correlated with the concentration of butyrate (*r* = 0.595, *p* = 0.041), whereas the relative abundance of *Prevotella_*1 negatively correlated with the concentration of butyrate (*r* = −0.742, *p* = 0.006). The relative abundance of *Candidatus_Saccharimonas* negatively correlated with the apparent digestibility of NDF (*r* = −0.583, *p* = 0.047).

## 4. Discussion

The particle size of feed affects DMI, the surface area attacked by microorganisms, and digestive passage of the digestive tract [22]. In this study, the DMI of the SL calves was higher than those of the ML and LL calves; therefore, our result is in agreement with the report that chopping the hay could increase the DMI of cattle [23]. Similarly, Castells et al. found that feeding pelleted starter together with chopped grass hay could increase the DMI of dairy calves [24]. In this study, chopping roughage had a quadratic effect on the OM apparent digestibility; the reason may be that the SL calves had the highest roughage intake and roughage ratio, while the LL claves were fed the highest roughage length; and diets with either higher roughage ratio or longer roughage length have longer rumination time and lower passage rate in the gastrointestinal, thereby resulting in high OM apparent digestibility [7].

It is generally believed that increasing the length of dietary roughage can increase the chewing activity, further stimulate the secretion of saliva, and eventually increase the rumen buffering capacity [9]. In this study, chopping roughage length did not affect the ruminal fluid pH; the reason may be that in this experiment the roughage lengths all exceeded 4 cm, the lengths were long enough to stimulate the chewing activity; and the ruminal pH is primary determined by the balance between the secretion of salivary buffers and the production of fermentation acids [25]. In agreement with our results, Kononoff et al. reported that the ruminal pH of lactating dairy cows fed with corn silage with lengths ranging from 7.4 to 8.8 mm were similar [26]. The VFAs are the main source of energy for ruminants, and of them, butyrate and propionate can promote rumen epithelium differentiation and rumen papillae development [14]. In this study, chopping roughage linearly and quadratically increased the concentration of rumen butyrate. Additionally, our results showed that the abundances of *Ruminococcaceae_UCG-005* positively correlated with rumen butyrate concentrations. In addition, chopping roughage increased the abundance of the genus *Pyramidobacter*, and it has been previously reported that its abundance positively correlates with the concentration of butyrate in the rumen of early starter feeding lambs [27], indicating that the increased rumen butyrate is due to the increase of butyrate-related bacteria in the rumen. Consistent with our results, Biddle et al. found that Ruminococcaceae is able to degrade cellulose and hemicellulose in gut environments, with the products including butyrate [28]. In this study, chopping roughage linearly increased the concentrations of rumen fluid isovalerate. Liu et al. found that isovalerate could switch the ruminal fermentation patterns into more butyrate production, verifying the same trend of ruminal butyrate and isovalerate observed in the present study [29]. Additionally, Cline et al. found a positive correlation between cellulolytic microorganisms and the rate of valerate utilization in rumen [30].

A positive correlation exists between cellulolytic microorganisms and valerate utilization in the rumen. In this study, chopping roughage linearly and quadratically increased the concentrations of rumen fluid valerate, suggesting that more abundances of cellulolytic bacteria colonized in the SL calves led to the increase in valerate. In this study, chopping roughage linearly increased the concentration of plasma glucose. Khan et al. found that greater starch consumption can trigger hepatic glucogenic activity and consequently greater glucose supply in calves, which results in greater glucose irreversible loss [31]. Hence, in the present study, the LL calves that were fed less roughage had a high concentration of starch in the rumen, possibly improving the availability and oxidation of glucose, which eventually resulted in reductions in plasma glucose concentrations. Plasma total protein, including immunoglobulins and non-immunoglobulins serum proteins, correlates with the immune system of animals [32]. In this study, the SL calves had higher concentrations of plasma total protein, suggesting that chopping roughage enhanced the immunity of weaned calves. Blood urea nitrogen concentration was previously found to have a positive relationship with CP intake and its ruminal degradability [33]. In this study, the roughage length had no effects on the plasma urea nitrogen; the result, which is consistent with the similar concentrations of NH_3_-N in rumen fluid, verified the view that the urea nitrogen usually reflects changes in ruminal NH_3_-N [34].

A calf’s gastrointestinal tract is predominated by a great diversity of microorganisms, which plays vital roles in fiber degradation and the morphological development of rumen [35]. Microorganisms including protozoa, anaerobic, bacteria, and fungi, are absent in the rumen of newborn calves [36]. Only a small number of microorganisms can survive, colonize, multiply, and ultimately form specific microflora in rumen with the growth of calves [37]. Bacteria alpha diversity is an indicator used to estimate the species richness and uniformity of a certain sample [38]. In this study, chopping roughage linearly or quadratically increased the diversity indices including chao 1, observed species, and richness estimator PD_whole_tree, indicating that roughage length affected the distribution of the rumen microflora by increasing the richness and uniformity of the rumen microbial community. In this study, consistent with the view that the rumen microbial ecosystem is dominated by a core community composed of microbes [39], the dominant phyla were Bacteroidetes and Firmicutes. We found that chopping roughage linearly and quadratically increased the abundances of *Synergistetes* and *Mogibacterium*, whose abundances are positively associated with the age of calves [40,41], indicating that chopping roughage at a short length could promote the process of bacterial colonization in the rumen of weaned calves. The family Ruminococcaceae can crack roughage cellulose and hemicellulose components due to a large number of endo-1, 4-beta-xylanase, cellulase genes, alpha-glucosidases, and galactosidases [28]. In this study, chopping roughage increased the relative abundances of Ruminococcaceae; the reason may be that the equal concentrate intake among the treatments and higher DMI in the SL claves resulted in a higher roughage content in the rumen. In addition, chopping roughage decreased the abundances of *Succinivibrio* and *Selenomonas_1*. Of them, *Succinivibrio* in the rumen of calves positively correlate with concentrate intake and starch digestion [42]. *Selenomonas* is an obligately saccharolytic bacteria that participate in the fermentation of soluble sugars and lactate in the rumen [43]. Therefore, the increased roughage length increased the relative percentage of concentrate, and eventually led to the increased abundances of these genera.

The genus *Pyramidobacter* positively correlate with the synthesis of thiamine, which could attenuate inflammation response by suppressing the expression of proinflammatory cytokines and decreasing ruminal lipopolysaccharide (LPS) levels [44]. In this study, the decreasing length of roughage increased the abundance of *Pyramidobacter*, suggesting that chopping roughage length is beneficial to the health of weaned calves. In addition, our results showed that decreasing the length of roughage increased the abundances of genera *Papillibacter* and *Eubacterium_hallii_group*, whose abundances affect butyrate production in the rumen [45,46,47], indicating that chopping roughage altered the rumen bacterial community and promoted the development of the rumen.

## 5. Conclusions

Chopping roughage promoted the process of bacterial colonization by increasing age-related bacteria *Synergistetes* and *Mogibacterium* improved the development of rumen by increasing the butyrate-related bacteria *Papillibacter* and *Eubacterium_hallii_group*, and increased the rumen butyrate concentration. Chopping roughage also altered plasma parameters, especially the total protein, which is beneficial to the subsequent health of calves. This study illustrated that chopping roughage at SL could improve the rumen bacterial community, alter rumen fermentation, and eventually promote the rumen development of weaned calves.

## Figures and Tables

**Table 1:** Ingredients and chemical composition of experimental diets.

Item	Starter Pellets	Oat Hay
Ingredient (g/kg, DM)		
Ground corn	350	
Soybean meal	50	
Corn gluten meal	80	
DDGS	150	
Soybean hulls	150	
Premix	50	
Molasses	20	
Salt	10	
Wheat bran	50	
Corn germ meal	40	
Wheat flour	50	
Chemical composition (g/kg, DM)		
OM	900.2	939.3
CP	228.3	65.4
EE	10.4	27.1
NDF	253.3	712.1
ADF	71.2	437.1

DM: dry matter; DDGS: distillers dried grains with soluble; OM: organic matter; CP: crude protein; EE: ether extract; NDF: neutral detergent fiber; ADF: acid detergent fiber.

**Table 2:** Effects of roughage length on the growth performance and nutrient apparent digestibility of weaned calves.

Item		Diet			SEM	*p*-Value	
		SL	ML	LL		Linear	Quadratic
	Growth performance						
Initial body weight (kg)		158.7	164.2	160.7	3.790	0.711	0.588
Final body weight (kg)		196.8	200.0	196.0	4.514	0.896	0.805
ADG (kg/d)		1.12	1.05	1.04	0.055	0.286	0.527
DMI (kg/d)	Starter pellets	2.50	2.50	2.50			
	Oat hay	1.74	1.51	1.47	0.017	0.001	<0.001
Roughage ratio (%)		41.0	37.6	37.0	0.645	0.001	<0.001
FCR		3.78	3.81	3.81	0.021	0.280	0.495
	Nutrient apparent digestibility (%)						
OM		86.13	81.76	83.59	1.147	0.149	0.037
CP		86.25	83.30	84.00	1.245	0.213	0.232
EE		83.39	82.07	85.26	1.861	0.483	0.485
NDF		84.26	81.57	82.20	1.418	0.312	0.384
ADF		84.04	80.99	82.54	1.282	0.421	0.256

SL: short length; ML: medium length; LL: long length; BW: body weight; ADG: average daily gain; DMI: dry matter intake; FCR: feed conversion ratio; OM: organic matter; CP: crude protein; EE: ether extract; NDF: neutral detergent fiber; ADF: acid detergent fiber.

**Table 3:** Effects of roughage length on rumen fermentation of weaned calves.

Item	Diet			SEM	*p*-Value	
	SL	ML	LL		Linear	Quadratic
pH	6.41	6.65	6.58	0.095	0.215	0.198
NH_3_-N (mg/dL)	7.42	6.39	6.12	0.618	0.191	0.389
VFA (mmol/L)						
TVFA	71.42	62.41	63.47	3.539	0.123	0.160
Acetate	48.21	42.22	43.37	2.455	0.175	0.202
Propionate	13.45	12.12	11.96	0.663	0.118	0.231
Isobutyrate	0.95	0.89	0.95	0.091	0.941	0.875
Butyrate	6.77	5.54	5.54	0.383	0.031	0.043
Isovalerate	1.31	1.10	1.08	0.084	0.062	0.116
Valerate	0.73	0.55	0.56	0.054	0.034	0.038
A/P	3.58	3.49	3.63	0.070	0.633	0.371

SL: short length; ML: medium length; LL: long length; VFA: volatile fatty acids; TVFA: total volatile fatty acid; A/P: the ratio of acetate to propionate.

**Table 4:** Effects of roughage length on the plasma parameters of weaned calves.

Item	Diet			SEM	*p*-Value	
	SL	ML	LL		Linear	Quadratic
Glucose (mmol/L)	4.864	4.703	4.462	0.137	0.043	0.129
Triglyceride (mmol/L)	0.304	0.314	0.281	0.025	0.508	0.626
Cholesterol (mmol/L)	3.214	2.803	2.452	0.159	0.002	0.007
Total protein (g/L)	69.74	64.13	65.54	1.314	0.039	0.013
Urea nitrogen (mmol/L)	5.057	5.409	5.487	0.201	0.135	0.285
INS (mIU/L)	26.81	33.59	19.99	4.417	0.300	0.109
GH (ng/mL)	5.576	5.407	6.737	0.485	0.103	0.124
IGF1 (ng/mL)	85.35	78.74	99.25	9.877	0.329	0.338

SL: short length; ML: medium length; LL: long length; INS: insulin; GH: growth hormone; IGF1: insulin-like growth factor 1.

**Table 5:** Effects of roughage length on the rumen bacteria alpha diversity of weaned calves.

Item	Diet			SEM	*p*-Value	
	SL	ML	LL		Linear	Quadratic
Chao1	1844.9	1671.2	1654.7	35.92	0.007	0.008
Observed_species	1440.5	1269.1	1304.5	41.20	0.070	0.038
PD_whole_tree	132.7	120.6	122.3	2.75	0.042	0.025
Shannon	8.60	8.22	8.31	0.152	0.214	0.224

SL: short length; ML: medium length; LL: long length.

## Supplementary Materials

The following are available online at https://www.mdpi.com/2076-2615/10/11/2149/s1, Table S1: Effects of roughage length on the phylum (as a percentage of the total sequences) of the ruminal bacterial community. Table S2: Effects of roughage length on the genus (as a percentage of the total sequences) of the ruminal bacterial community.
