# Supplementary material for: Chopping Roughage Length Improved Rumen Development of Weaned Calves as Revealed by Rumen Fermentation and Bacterial Community
Source: Animals (Basel). 2020 Nov 19;10(11):2149. doi: 10.3390/ani10112149 (PMC7699230; doi:10.3390/ani10112149)
Supplement: Supplementary file 1 [file animals-10-02149-s001.pdf]

Chopping roughage length improved rumen development of weaned calves as revealed by rumen fermentation and bacterial community

Haibo Wang [1,2], Fei Wu [1], Tianci Guan [1], Yangxiang Zhu [1], Zhantao Yu [3], Depeng Zhang [1], Siyu Zhang [1], Huawei Su [1,*], Binghai Cao [1,*]

[1] State Key Laboratory of Animal Nutrition, College of Animal Science and Technology, China Agricultural University, Beijing, 100193, People's Republic of China

[2] Key Laboratory of Qinghai-Tibetan Plateau Animal Genetic Resource Reservation and Utilization (Southwest Minzu University), Ministry of Education, Chengdu, 610000, People's Republic of China

[3] Department of Animal Science, University of Tennessee, Knoxville, TN, 37996, USA

[*] **Correspondence:** caucaobh@163.com (B.C.), suhuawei@cau.edu.cn (H.S.). Tel./Fax: +86-010-6281-4346 (B.C.)

Supplementary Table S1

Effects of roughage length on the phylum (as a percentage of the total sequences) of the ruminal bacterial community.

Item	Diet			SEM	*P*-value	
	SL	ML	LL		Linear	Quadratic
Bacteroidetes	46.10	48.64	52.17	3.299	0.201	0.458
Firmicutes	37.18	33.76	33.80	2.125	0.274	0.458
Proteobacteria	5.47	5.40	4.12	0.763	0.229	0.411
Tenericutes	1.82	1.88	2.15	0.296	0.436	0.718
Saccharibacteria	1.61	1.67	1.72	0.271	0.759	0.956
Fibrobacteres	1.50	2.11	1.03	0.324	0.404	0.115
Spirochaetae	1.42	1.61	1.44	0.248	0.961	0.835
Cyanobacteria	1.34	1.62	0.73	0.420	0.335	0.351
Verrucomicrobia	1.15	1.08	0.74	0.276	0.297	0.549
SR1_Absconditabacteria	0.70	1.01	1.00	0.185	0.255	0.425
Actinobacteria	0.57	0.45	0.49	0.075	0.438	0.517
Elusimicrobia	0.41	0.26	0.19	0.100	0.139	0.331
Lentisphaerae	0.28	0.24	0.18	0.056	0.231	0.504
Synergistetes	0.23	0.14	0.10	0.029	0.010	0.036

SL, short length; ML, medium length; LL, long length.

Supplementary Table S2

Effects of roughage length on the genus (as a percentage of the total sequences) of the ruminal bacterial community.

Item	Diet			SEM	*P*-value	
	SL	ML	LL		Linear	Quadratic
Unidentified	25.86	24.38	23.55	1.951	0.400	0.709
Prevotella_1	16.52	23.94	25.16	3.903	0.138	0.288
Rikenellaceae_RC9_gut_group	6.42	4.20	4.53	0.860	0.159	0.199
Christensenellaceae_R-7_group	5.28	3.95	5.33	0.428	0.945	0.081
Ruminococcaceae_NK4A214_group	3.55	3.25	3.23	0.409	0.576	0.831
Succiniclasticum	2.39	3.39	2.68	0.556	0.724	0.453
Prevotellaceae_UCG-001	2.79	1.96	2.86	0.381	0.911	0.233
Succinivibrionaceae_UCG-002	2.95	2.29	2.29	0.449	0.303	0.511
Prevotellaceae_UCG-003	1.83	1.87	1.93	0.242	0.775	0.961
Ruminococcus_2	1.96	1.67	1.48	0.421	0.416	0.728
Candidatus_Saccharimonas	1.61	1.67	1.72	0.271	0.759	0.956
Fibrobacter	1.49	2.10	1.03	0.324	0.411	0.116
Ruminococcaceae_UCG-014	1.45	1.59	1.33	0.222	0.707	0.726
Butyrivibrio_2	1.45	1.14	1.58	0.205	0.689	0.342
Treponema_2	1.21	1.45	1.31	0.260	0.788	0.813
Ruminococcaceae_UCG-005	1.36	1.23	0.86	0.153	0.040	0.112
Lachnospiraceae_NK3A20_group	1.27	1.00	1.13	0.199	0.634	0.646
Ruminococcus_1	0.97	1.31	0.92	0.218	0.883	0.427

Pseudobutyrivibrio	0.79	0.68	1.12	0.170	0.214	0.217
Anaeroplasma	0.66	0.92	0.92	0.226	0.417	0.656
Ruminococcaceae_UCG-010	1.02	0.76	0.73	0.100	0.072	0.140
Veillonellaceae_UCG-001	0.78	0.77	0.72	0.122	0.715	0.932
Saccharofermentans	0.77	0.72	0.73	0.110	0.777	0.932
Eubacterium_coprostanoligenes_group	0.75	0.73	0.69	0.077	0.565	0.852
Erysipelotrichaceae_UCG-004	0.60	0.53	0.80	0.120	0.260	0.283
Lachnospiraceae_XPB1014_group	0.58	0.49	0.49	0.044	0.200	0.324
Succinivibrio	0.25	1.17	0.12	0.272	0.814	0.045
Prevotellaceae_UCG-004	0.77	0.33	0.36	0.111	0.037	0.036
Acetitomaculum	0.45	0.47	0.48	0.084	0.821	0.975
Papillibacter	0.60	0.42	0.31	0.082	0.025	0.089
Prevotellaceae_NK3B31_group	0.52	0.33	0.47	0.060	0.643	0.111
Lachnospiraceae_AC2044_group	0.43	0.46	0.36	0.089	0.581	0.753
Unidentified_rumen_bacterium_RFN46	0.72	0.21	0.31	0.230	0.246	0.306
Ruminobacter	0.50	0.35	0.29	0.124	0.244	0.503
Lachnospiraceae_ND3007_group	0.36	0.32	0.41	0.131	0.802	0.895
Eubacterium_ruminantium_group	0.32	0.36	0.37	0.055	0.503	0.792
Moryella	0.37	0.29	0.34	0.065	0.672	0.670
Desulfovibrio	0.39	0.26	0.31	0.045	0.312	0.181
Ruminococcus_gauvreauii_group	0.25	0.32	0.33	0.026	0.068	0.153
Anaerovorax	0.34	0.27	0.22	0.040	0.052	0.163
Phocaeicola	0.28	0.23	0.29	0.040	0.893	0.579
Family_XIII_AD3011_group	0.35	0.19	0.21	0.038	0.055	0.034
Prevotellaceae_Ga6A1_group	0.22	0.22	0.23	0.059	0.859	0.980
Probable_genus_10	0.16	0.20	0.20	0.044	0.525	0.786
Anaerotruncus	0.19	0.18	0.15	0.030	0.337	0.595
Elusimicrobium	0.24	0.16	0.09	0.074	0.166	0.403
Ruminococcaceae_UCG-002	0.19	0.15	0.14	0.036	0.345	0.613
Selenomonas_1	0.11	0.28	0.10	0.043	0.907	0.025
Prevotellaceae_YAB2003_group	0.11	0.19	0.17	0.054	0.439	0.555
Senegalimassilia	0.15	0.13	0.16	0.014	0.463	0.361
Uncultured	0.05	0.27	0.10	0.094	0.786	0.273
Anaerovibrio	0.17	0.11	0.13	0.026	0.269	0.305
Oribacterium	0.11	0.14	0.15	0.028	0.347	0.656
Roseburia	0.13	0.15	0.12	0.053	0.873	0.916
Atopobium	0.14	0.12	0.13	0.036	0.896	0.926
Ruminococcaceae_UCG-013	0.16	0.18	0.05	0.071	0.318	0.424
Blautia	0.11	0.11	0.17	0.020	0.099	0.119
Defluviitaleaceae_UCG-011	0.12	0.10	0.14	0.024	0.532	0.455
Candidatus_Endomicrobium	0.17	0.10	0.10	0.032	0.125	0.218
Eubacterium_ventriosum_group	0.14	0.08	0.13	0.018	0.648	0.121
Olsenella	0.14	0.11	0.10	0.029	0.355	0.658
Eubacterium_hallii_group	0.15	0.08	0.11	0.016	0.246	0.034

Ruminiclostridium_6	0.13	0.10	0.10	0.022	0.261	0.457
Ruminococcaceae_V9D2013_group	0.15	0.08	0.10	0.040	0.344	0.457
Pyramidobacter	0.15	0.11	0.06	0.018	0.005	0.024
Mogibacterium	0.14	0.06	0.12	0.016	0.613	0.014
SP3-e08	0.09	0.07	0.15	0.025	0.192	0.123
Marvinbryantia	0.12	0.08	0.11	0.019	0.965	0.352

SL, short length; ML, medium length; LL, long length.
